# Evaluation of rational drug use based on World Health Organization core drug use indicators in selected public hospitals of eastern Ethiopia: a cross sectional study

**DOI:** 10.1186/s12913-017-2097-3

**Published:** 2017-02-23

**Authors:** Mekonnen Sisay, Getnet Mengistu, Bereket Molla, Firehiwot Amare, Tesfaye Gabriel

**Affiliations:** 1 Department of Pharmacology, School of Pharmacy, College of Health and Medical Sciences, Haramaya University, P.O. Box 235, Harar, Ethiopia; 2 Department of Clinical Pharmacy, School of Pharmacy, College of Health and Medical Sciences, Haramaya University, P.O. Box 235, Harar, Ethiopia; 3 Department of Pharmaceutics and Social Pharmacy, School of Pharmacy, College of Health Sciences, Addis Ababa University, P.O. Box 1176, Addis Ababa, Ethiopia

**Keywords:** Rational drug use, World Health Organization, Prescribing indicators, Patient care indicators, Health facility indicators

## Abstract

**Background:**

Despite the complexity of drug use, a number of indicators have been developed, standardized and evaluated by the World Health Organization (WHO). These indicators are grouped in to three categories namely: prescribing indicators, patient care indicators and facility indicators. The study was aimed to evaluate rational drug use based on WHO-core drug use indicators in Dilchora referral hospital, Dire Dawa; Hiwot Fana specialized university hospital, Harar and Karamara general hospital, Jigjiga, eastern Ethiopia.

**Methods:**

Hospital based quantitative cross sectional study design was employed to evaluate rational drug use based on WHO core drug use indicators in selected hospitals. Systematic random sampling for prescribing indicators and convenient sampling for patient care indicators was employed. Taking WHO recommendations in to account, a total of 1,500 prescription papers (500 from each hospitals) were investigated. In each hospital, 200 outpatient attendants and 30 key essential drugs were also selected using the WHO recommendation. Data were collected using retrospective and prospective structured observational check list. Data were entered to EPI Data Version 3.1, exported and analyzed using SPSS version 16.0. Besides, the data were evaluated as per the WHO guidelines. Statistical significance was determined by one way analysis of variance (ANOVA) for some variables. *P*-value of less than 0.05 was considered statistically significant. Finally, tabular presentation was used to present the data.

**Results:**

Mean, 2.34 (±1.08) drugs were prescribed in the selected hospitals. Prescriptions containing antibiotics and that of injectables were 57.87 and 10.9% respectively. The average consultation and dispensing time were 276.5 s and 61.12 s respectively. Besides, 75.77% of the prescribed drugs were actually dispensed. Only 3.3% of prescriptions were adequately labeled and 75.7% patients know about the dosage of the prescription. Not more than, 20(66.7%) key drugs were available in stock while only 19(63.3%) of key drugs had adequate labeling. On average, selected key drugs were out of stock for 30 days per year. All of the hospitals included in the study used the national drug list, formulary and standard treatment guidelines but none of them had their own drug list or guideline.

**Conclusion:**

Majority of WHO stated core drug use indicators were not met by the three hospitals included in the study.

## Background

Rational drug use (RDU) generally covers appropriate prescribing, appropriate dispensing and appropriate patient use of medicines for the diagnosis, prevention, mitigation and treatment of diseases. RDU can also be described as safe, cost-effective and economically viable use of drugs. To ehnance RDU, the patient should receive medicines appropriate to their heath care conditions, at optimum doses and sufficient time, as well as at the cost that the individual and the community can afford [1]. The ultimate goal of RDU is to foster better quality of pharmaceutical care, to minimize the cost of drug therapy, to avoid preventable adverse drug reactions and drug interactions, to maximize therapeutic outcomes and to promote patient adherence [2, 3].

WHO developed core and complementary drug use indicators for evaluation of drug use in healthcare settings. Among which, the core drug use indicators have been considered as the first line indicators validated by WHO for measurement of drug use. The core drug use indicators are more informative, more feasible, less likely to fluctuate over time and place as well as easier to measure drug use than the complementary indicators [4, 5]. Therefore, the core indicators have been selected for better quantitative evaluation of RDU. There are three major categories of core drug use indicators namely, prescribing indicators (average number of drugs per encounter; percentage of drugs prescribed with generic name; percentage of encounters with antibiotics prescribed, percentage of encounters with injections prescribed and percentage of drugs prescribed from EDL) patient care indicators (average consultation time, average dispensing time, percentage of drugs actually dispensed, percentage of drugs actually labeled and patient knowledge of how to take the drug), and health facility indicators (availability of essential drugs, availability of STGs, formularies and EDLs) [6, 7].

Pharmaceutical expenditure is up to 70/75% of total healthcare expenditure in low and middle-income countries [8]. However, irrational use of drugs has been primarily observed in healthcare systems of developing countries. WHO estimates that more than half of all drugs are irrationally prescribed or dispensed and more than half of the patients fail to adhere the prescribed regimens [5]. Common reasons for irrational use of medicines include:- lack of adequate information about the prescribed drugs, faulty and inadequate training of medical graduates, poor communication between health care providers and patients, lack of diagnostic facilities, demand from the patient (assuming that ‘every ill has a pill’), and defective drug supply system [9].

Irrational use of drugs can have a significant adverse effect on health care costs, quality of pharmaceutical care and emergence of antimicrobial resistance. Other negative impacts are the increased risk of adverse drug reactions, drug-drug interactions and non-adherence of patients to the treatment [9–12].

The information available suggests medicines are not optimally used. This inappropriate drug use has serious health and economic influences for the success of national health care system. The irrational use of medicines is a worldwide problem increasing morbidity, mortality and costs through increasing adverse drug reactions and hence patients are not achieving their desired outcomes [13, 14].

By and large, the causes of irrational drug use are highly vexed and complicated involving the healthcare system, the supply system, the health professionals, the patient and the community at large. Considering such problem, the overall drug use pattern should be evaluated to put a baseline data for further in-depth investigation of drug use to probe the underlying causes and interventional strategies to be implemented so as to try and reverse worrying trends in drug utilization in Ethiopia. Therefore, the study is, aimed to evaluate RDU by using WHO core drug use indicators in selected public hospitals of eastern Ethiopia.

## Methods

### Study design and setting

Facility based quantitative cross sectional study design was employed to evaluate rational drug use based on WHO core drug use indicators in the Dilchora referral hospital (DRH), Dire Dawa; Hiwot Fana specialized University hospital (HFSUH), Harar and Karamara general hospital (KGH), Jigjiga,. The study was targeted at three tertiary care public hospitals found in two regions and one city administration (Dire Dawa) of eastern Ethiopia. The study was conducted from Sep 1 to Nov 30, 2014. Retrospective cross sectional study design was used to evaluate prescribing indicators while prospective cross-sectional design was employed for patient care and facility indicators.

### Study population

All outpatient prescriptions dispensed from Jan 1, 2014 to Jun 30, 2014 (prescribing indicators); patient attendants and their prescriptions in the outpatient departments (OPDs) of selected hospitals from Sep 1 to Nov 30, 2014 (patient care indicators) and drugs under essential drug lists (EDL) of 2013 of Ethiopia were included. However, prescriptions that contain only medical supplies like glove, syringe and patient attendants outside the normal working hours were excluded.

### Sample size determination and sampling technique

#### For prescribing indicators

Considering WHO recommendation, 500 prescribing encounters were taken from each corresponding hospital [7]. So, a total of 1,500 prescription papers were investigated in the study. A systematic random sampling technique was employed to select 500 outpatient prescriptions from each hospital by taking every ten prescription in DRH, every eight in KGH and HFSUH.

#### Patient care indicators

Based on WHO criteria, at least 100 sample sizes of outpatient attendants (encounters) are recommended in individual health facility [7]. Therefore, to get a more reliable result (greater point estimate of the population) 200 samples of patients were assessed in each hospital. The patient attendants with their prescriptions in OPDs were sampled by convenient sampling technique prospectively.

#### For health facility indicators

Thirty key drugs were selected from each hospital as per WHO recommendation which is a minimum of 15 essential drugs in each health facility [7]. These key drugs being used for the management of top ten diseases of the respected hospitals were selected by communicating with prescribers and dispensers and reviewing national guideline.

### Data collection instrument and techniques

Data were collected using structured observational check list for prescribing, patient care and health facility indicators. A reference of patient prescription papers was used to check the patient knowledge of how to take the correct dosage. Stop watch was used to determine the contact time of health care providers with patient (consultation and dispensing time).

Data regarding prescribing indicators were taken from sampled prescription records retrospectively and filled in structured check list accordingly by careful observation. On the other hand, data regarding patient care indicators was taken from patient attendants and their prescriptions in OPD during the period of data collection prospectively and was recorded in observational check list. Among patient care indicators, data regarding patient knowledge of how to take correct dosage was collected through face to face interview and recorded as 1 or 0 for each patient (all-none principle) as per the guideline. Besides, the availability of key/essential drugs, EDL and STG was assessed in OPD, and was filled in facility indicator form accordingly.

### Data quality control

To ensure the data quality, the data collectors (pharmacy technicians) and supervisors (pharmacists) were trained by the principal investigators for three days. The English version of the checklist was translated to Amharic, Afaan Oromo and Somali languages and then back to English to maintain its consistency for data collection purpose. The data collection instruments were pre-tested in Jugal hospital, Harar by two co-investigators of the study. Completeness and consistency of the collected data was ensured by making frequent checks on the data collection process.

### Data processing and analysis

Data was entered to EPI Data Version 3.1. Then, it was exported and analyzed using SPSS version 16.0. Besides, the data were evaluated as per the WHO guidelines. Statistical significance was determined by one way analysis of variance (ANOVA) in some of the patient care indicators. P-value less than 0.05 was considered to be statistically significant. Finally, tabular presentation was used to present the data.

## Results

### Prescribing indicators

For the assessment of WHO prescribing indicators, a total of 1500 prescription encounters (500 in each hospital) were included in the study making 100% completion and response rates. On average 2.34 (±1.08) drugs were prescribed in selected public hospitals of eastern Ethiopia and 39.3% of the prescription contains three or more drugs (Table 1). Besides, from the total of 1500 prescription encounters, 3174 (90.61%) of the drugs were prescribed by generic name with the lowest value observed in HFSUH (85.04%) (Table 2). Amongst the prescription encounters assessed, the study revealed that 868 (57.87%) prescriptions contained antibiotics. Moreover, 163 (10.9%) prescription contained at least one injectable medication (Table 3). Amongst antibiotics prescribed, 44.4% of them were amoxicillin or amoxicillin + clavulanic acid followed by ciprofloxacin (15.4%) (Table 4).

**Table 1:** Number of drugs per prescribing encounters (degree of polypharmacy) in eastern Ethiopia selected hospitals (DRH, HFSUH and KGH), Jan 1, 2014 - Jun 30, 2014 (*n* = 1500)

Number of drugs	DRH	HFSUH	KGH	Overall result	WHO standard
One	147(29.4)	108(21.6)	96(19.2)	351(23.4)	
Two	197(39.4)	184(36.8)	178(35.6)	559(37.3)	
Three	137(27.4)	116(23.2)	144(28.8)	397(26.5)	
Four	17(3.4)	55(11.0)	70(14)	142(9.5)	
Five	1(0.2)	25(5)	10(2)	36(2.4)	
Six	____	10(2)	____	10(0.7)	
Seven or more	1(0.2)	2(0.4)	2(0.4)	5(0.3)	
Average value	2.06 (± 0.877)^a^	2.49 (±1.235)^a^	2.46 (± 1.056)^a^	2.34 (±1.08)^b^	≤ 2 (1.6–1.8)

^a^Average value per hospital; ^b^overall average number of drugs per encounter

**Table 2:** Percentage of drugs prescribed by generic name in DRH, HFSUH and KGH, eastern Ethiopia, Jan 1, 2014–Jun 30, 2014 (*n* = 1500 encounters)

Hospital name	# of drugs prescribed	# drugs prescribed by generic name	% drugs prescribed by generic name	WHO standard
DRH	1032	984	95.35^a^	
HFSUH	1243	1057	85.04^b^	
KGH	1228	1133	92.26^a^	
Over all	3503	3174	90.61	100%

^a^ Mild deviation ^b^ moderate deviation

**Table 3:** Percentages of encounters with antibiotics and injections prescribed in DRH, HFSUH and KGH, eastern Ethiopia, Jan 1, 2014–Jun 30, 2014 (*n* = 1500)

Prescribing indicators		DRH	HFSHU	KGH	Over all	WHO standard
Percentage of encounters with antibiotics	Total number of encounters	500	500	500	1500	
	Encounters with antibiotic	271	250	347	868	
	% of encounters with antibiotics	52.2	50	69.4	57.87^a^	< 30 (20–26.8%)
Percentage of encounters with injections	Encounters with injections	52	93	18	163	
	% of encounters with injections	10.4	18.6	3.6	10.9	(13.4–21.1%)

^a^Significant deviation from WHO criteria

**Table 4:** Antibiotics prescribed in DRH, HFSUH and KGH, eastern Ethiopia, Jan 1, 2014–Jun 30, 2014 (*n* = 1500)

Antibiotics	Frequency (%)			Over all
	DRH	HSUH	KGH	
Amoxicillin/ amoxicillin + clavulinic acid	130(42.9)	120(46.0)	165(44.5)	415(44.4)
Ciprofloxacin	40(13.2)	36(13.8)	68(18.3)	144(15.4)
Doxycycline	23(7.6)	21(8.0)	30(8.1)	74(7.9)
Norfloxacin	23(7.6)	26(10.0)	8	57(6.1)
Cotrimoxazole	7	8	30(8.1)	45(4.8)
Cloxacillin	14(4.6)	12(4.6)	12(3.2)	38(4.1)
Erythromycin	8	2	20(5.4)	30(3.2)
Benzanthine Penicillin G	10(3.3)	12(4.6)	6	28(3.0)
Chloramphenicol	7	5	13(3.5)	25(2.7)
Cephalexin	12(4.0)	5	3	20(2.1)
Clarithromycin	4	1	9	14(1.5)
Gentamicin	5	4	3	12(1.3)
Ampicillin	4	3	4	11(1.2)
Tetracycline	3	5	-	8
Tetracort	8	-	-	8
Chloramphenicol eye ointment	5	-	-	5
Azithromycin	-	1	-	1
Total	303(100)	261(100)	371(100)	935(100)

### Patient care indicators

The average consultation time and dispensing time in selected public hospitals of eastern Ethiopia were found to be 4.61 min and 61.12 s, respectively. Besides, on average, only 75.77% of prescribed drugs were actually dispensed in selected hospitals with lowest value seen in HFSUH (69.27%) (Table 5) and among dispensed prescriptions within the health facilities, only 3.3% of them were adequately labeled in target hospitals when taken together with zero value recorded in KGH (Table 6). Moreover, 75.7% patients know about the correct dosage schedule of the prescription in selected public hospitals of eastern Ethiopia (Table 7).

**Table 5:** Consultation time, dispensing time and percentage of drugs actually dispensed in DRH, HFSUH and KGH, eastern Ethiopia, Sep1-Nov 30, 2014 (*n* = 600)

Patient care indicators	DRH	HFSUH	KGH	Overall	WHO standards	*P*-value
Average consultation time (minute)	4.27 (±117.64)	6.36 (±266.22)	3.20(±83.88)	4.61(±191.58)	10 min	*P* < 0.001*
Average dispensing time (Sec)	88.62(±62.56)	37.58 (±30.35)	57.16(±40.78)	61.12(±51.01)	>180 s	*P* < 0.001*
Total number of drugs prescribed	405	410	485	1300		
Total number of drugs dispensed	325	284	376	985		
Percentage of drugs actually dispensed	80.25	69.27	77.53	75.77	100%	*P* < 0.001*

*One way analysis of variance (ANOVA), *p* value less than 0.05 was considered to be statistically significant

**Table 6:** Labeling status of dispensed drugs in DRH, HFSUH and KGH, eastern Ethiopia, Sep1-Nov 30, 2014 (*n* = 600)

Hospital name	Contents of the label					Overall labeling status
	Patient Name	Drug strength	Drug dosage	Frequency of administration	Duration of treatment	
DRH	23(11.5%)	26(13.0%)	22(11.0%)	32(16.0%)	22(11.0%)	18(9.0%)^a^
HFSUH	4(2%)	11(5.5%)	13(6.5%)	15(7.5%)	5(2.5%)	2(1%) ^a^
KGH	0	2(1%)	21(10.5%)	24(12.0%)	17(8.5%)	0^a^
Over all	27(4.5%)	39(6.5%)	56(9.3%)	71(11.8%)	44(7.3%)	20(3.3%)

^a^ Major deviation from WHO set point

**Table 7:** Patient knowledge about dosage of dispensed drugs in DRH, HFSUH and KGH, eastern Ethiopia, Sep1-Nov 30, 2014 (*n* = 600)

Hospital name	Patient knowledge			Overall dosage schedule
	Dose	Frequency	Duration	
DRH	185(92.5%)	181(90.5%)	174(87.0%)	164(82.0%)^a^
HFSUH	179(89.5%)	186(93.0%)	144(72.0%)	134(67.0%)^a^
KGH	161(80.5%)	164(82.0%)	160(80.0%)	156(78.0%)^a^
Over all	525(87.5%)	531(88.5%)	478(79.7%)	454(75.7%)

^a^ Below WHO recommendations (ideal standard)

### Health facility indicators

Only 20(66.7%) key essential drugs were available in stock during the study and only 19(63.3%) of key drugs selected had adequate labeling. On average, selected key drugs were out of stock for 30 days per year (Table 8). All of the hospitals included in the study use the national EDL, formulary and STGs but none of them had their own EDLs, formularies or STGs (Table 9).

**Table 8:** Key essential drugs selected in DRH, HFSUH and KGH, eastern Ethiopia, Sep1-Nov 30, 2014 (*n* = 30)

Lists of key drugs	DR H	HFSUH	KGH
Amoxicillin capsule	✓	✓	✓
Artemether + lumefantrine (Quartem)	✓		✓
Ceftriaxone injection		✓	✓
Cimetidine			✓
Chloramphenicol			✓
Ciprofloxacin tablet	✓	✓	✓
Cloxacillin capsule		✓	
Diclofenac injection	✓	✓	
Diclofenac tablet		✓	✓
Doxycycline			✓
Enalapril	✓		
Erythromycin			✓
Ferrous sulfate			✓
Fluoxetine	✓		
Glibenclamide	✓		
Haloperidol	✓		
Metformin	✓		
Metoprolol	✓		
Metronidazole		✓	✓
Norfloxacin tablet	✓	✓	
NPH insulin	✓		
Omeprazole	✓	✓	✓
Oral rehydration salt (ORS)		✓	
Paracetamol		✓	
Regular insulin	✓		
RH(Rifampin + Isoniazid)		✓	✓
RHZE(Rifampine + Isoniazide + Pyrazineamide + Etambutol)		✓	✓
Sulphametoxazole + trimetoprime(cotrimoxazole)		✓	✓
Tetanus antitoxoid		✓	
Terra cortil®(oxytetracycline, hydrocortisone and polymyxin B sulfate)	✓		
Percentage of essential drugs	50^a^	50^a^	50^a^
WHO standard	100%		

^a^Deviation from WHO essential drug criteria by half

**Table 9:** Availability, recording and stock out of key drugs in DRH, HFSUH and KGH, eastern Ethiopia, Sep 1–Nov 30, 2014

Health facility indicators	DRH	HFSUH	KGH	Over all
Percentage in stock of key drugs	12(80.0%)^a^	6(40.0%)^a^	11(73.3%)^a^	20(66.7%)
% of drugs with adequate recording	11(73.3%) ^a^	0^a^	12(80.0%)^a^	19(63.3)
Average number of stock out days during the year for adequately recorded drugs	9.78	__	35^a^	30
Percentage of Expired drug	0	7(46.7%)^a^	6(40.0%)^a^	8(26.7%)^a^

^a^ Violates WHO indicators set for every health care setting

## Discussion

### Prescribing indicators

In this study, the average number of drug per prescription was 2.34 (2.06–2.49) with the highest value seen in HFSUH (2.49). The average number of drugs per prescription in the present study was slightly higher than the ideal WHO standard which is less than two (1.6–1.8) [7]. Besides, 39.3% of prescriptions contained three or more drugs. However, lower average numbers of medicines per encounter (1.9, 1.7, 1.8, 1.8, 2.1 and 2.2) were reported in different areas of Ethiopia such as Hawassa University Teaching and Referral Hospital (HUTRH) [14], southern Ethiopia [15] Jimma University Specialized Hospital (JUSH) [16], Dessie Referral Hospital [17], four selected hospitals of west Ethiopia [18] and in eight health facilities of Somali zone of eastern Ethiopia [19], respectively. Similarly, other studies outside of Ethiopia also reported a low number of drugs per encounter, for example, 1.4 in Sudan [20] and 1.3 in Zimbabwe healthcare settings [21]. Most of these values will fall within the WHO standard [7]. The degree of poly pharmacy was the same with the result obtained from Bule Hora Hospital, Southern Ethiopia where the average value was found to be 2.33 [22]. On the contrary, compared to this study, higher average number of drugs per encounter was also reported in different areas of the world such as 2.7 and 3.7 in Indian health care settings [23, 24], 2.8 in Nigerian army hospital [25]. Even though there are no adequate data that identify the underlying causes of poly-pharmacy in the study area, it might be related to lack of adequate knowledge and training of health professionals, variation in the health care delivery system, empirical prescribing and symptomatic treatment approaches, differences in socioeconomic status as well as morbidity and mortality characteristics of the population [14]. Generally, over prescribing might, in part, be attributable to drug-drug interactions, high risk of adverse drug reactions, wastage of drugs (extravagancy), and increased out-of-pocket expenditures for patients, among others [7].

The percentage of drugs prescribed by the generic name was found to be 90.61% (85.04–92.26%) with the lowest value recorded in HFSUH (85.04%). This result was somewhat lower than the ideal WHO standard (100%) [7]. Similarly, the current finding was lower than the findings of Gondar University Hospital (GUH) [26] and HUTRH [14], and health centers in Somali zone of eastern Ethiopia [19]. However, it was higher than studies undertaken in JUSH [16] and southern Ethiopia [15]. In addition, lower values of generic prescribing practices were recorded in several health care settings such as selected hospitals of west Ethiopia (79.2%) [18], Nigerian army hospitals (49.3%) [25], secondary care referral hospital of south India (42.9%) [23]. The high level of generic prescription could probably be attributed to the fact that the study was conducted in governmental hospitals, where procurement of generic drugs is the prevailing practice. Brand prescribing is associated with unnecessary treatment costs, difficulty of remembering the medication, accessibility and bioequivalence problems [7]. Therefore, more effort is to be invested to effectively avoid the problems of brand prescribing and to promote safe, cost effective and accessible generic drugs.

Percentage of prescribing encounters with antibiotic was 57.87% (50–69.4%) which is almost twice of the WHO standard (20–26.8%) [7]. The highest percentage of antibiotic encounter was recorded in KGH (69.4%). This finding was also higher than the finding of similar studies conducted in India [27], Nigeria [25], and different parts of Ethiopia [15–18] and almost similar with research done in HUTRH [14]. In the medicine use pattern study in 12 developing countries, the percentage of encounters with antibiotic prescribed were 63% in Sudan [20], 56% in Uganda [28], 48% in Nigeria [29] and 29% in Zimbabwe [21], among others.

Amoxicillin/amoxicillin + clavulanic acid was the most frequently prescribed antibiotic followed by ciprofloxacin. Similar study undertaken in different health care settings of Ethiopia found that amoxicillin to be the most frequently prescribed antibiotic in outpatient departments [14, 17, 19]. Inappropriate and over use of antibiotics, as observed in this study, might result in the emergence of antimicrobial resistance which is one of the major bottlenecks of chemotherapy for our globe [7]. If irrational use of antimicrobial agents is continued in this manner, the post antimicrobial era, where all the existing antimicrobial agents can be historical, will be expected in the near future. This is due to the imbalance between alarming rate of antimicrobial resistance and decelerating rate of new antimicrobial drug development [7].

On average, the percentage of encounters with injections prescribed was 10.7% in the present study with a high (18.6%) injection exposure recorded in HFSUH. In this study, the injection practices, in the outpatient settings, are generally acceptable. The current finding was better than studies conducted in India [24] Zimbabwe [21], southern Ethiopia [15], GUH [26] and HUTRH [14], west Ethiopia [18] and lied within WHO standard [7] but higher than the finding from JUSH [16]. The lower value in this study might be due to the fact that, as per the recommendation of WHO, only outpatient (ambulatory) patients’ prescription was taken with a great care so as not to overestimate the actual practice because of addition of inpatient prescription where prescribing injection is a common phenomena. Over use of injections may be associated with unnecessary injection related cost, risk of transmitting potential infections through needle stick injury and physiological and psychological pain during injection, difficulty of titrating overdose [7]. Therefore, safer, cost effective and simple oral alternatives should be promoted.

### Patient care indicators

The average consultation time in this study was 4.61 min which was considered to be short (less than 10 min as per the WHO standard) [7]. The average consultation time in HFSUH (6.36 min) is better than the other hospitals involved in the study. The result was similar to the finding of a research conducted in Eritrea (4.7 min) [30] but lower than on time spent for consultation in Nigeria [29]. Generally, longer consultation time had improved patient satisfaction and more effective resource use [9]. On average, 61.12 s was the average dispensing time calculated from this study. Time spent for dispensing was very short when compared to the average dispensing time for ten countries (105 s) [9, 31, 32] and from a similar study done in Zimbabwe (150 s) [33] and Nigeria (210 s) [34], India [23] and different healthcare settings of Ethiopia [19, 35]. This difference might be ascribed to variation on patient load on the individual health care settings. Very short dispensing time will negatively affect appropriate labeling and information provision about medications. Better average dispensing time was recorded in DRH.

From the prescribed medications, only 75.77% was actually dispensed: 80.25% DRH, 77.53% KGH and 69.27% HFSUH. This finding was lower than the average of 12 countries (89%) [9, 31, 32]. The difference might be partly ascribed to differences in the national logistic management system. This figure indicated that patients were prone for unnecessary medication charge by private drug retail outlets where margin of benefit might reach more than 100%.

Only 20 (3.3%) of medications were adequately labeled (DRH, 9%, HFSUH, 1%, and KGH, 0%) and 75.7% of patients had adequate knowledge about the dosage of their treatment. Patient knowledge was higher than similar study done in Zimbabwe (70%) [33]. The patient knowledge might look better in this study despite the low level of labeling practice but the figure was very low when looked at the potential impact of missing the dosage regimen on the rest 25% patients’ health outcome. Since the assessment of knowledge was done immediately, the impact of labeling on patient knowledge might seem insignificant. But, their knowledge will fade when the time elapses.

### Health facility indicators

None of the hospitals involved in the current study had its own EDL or STG. Since the prevalence of infectious and non-infectious diseases is affected by weather condition and lifestyle of the residents, hospital level EDL and STG are mandatory. Only 66.7% of key drugs were in stock while 63.3% of key drugs were adequately recorded. The finding is much lower than a result from Nigeria (83.3%) [36]. The absence of key drugs in stock was a strong indicator of weak pharmacy service in the hospitals and impair the overall services provided by the health sectors. Lack of adequate recording might have negatively affected the logistics management system and prone medications for theft. The problem is highly significant in HFSUH where none of the key drugs were recorded and 40% of the key drugs were available in stock. The average number of stock out days per year for adequately recorded drugs was 30 days. Percentage of expired drugs was 26.7% which is very significant for a poor country like Ethiopia where majority of the population have low access for essential drugs and majority of the drugs are imported with foreign currency at minimum tax.

### Limitation of the study

Generally, this study tried to address almost all WHO approved core drug use indicators in selected hospitals. These indicators highlight major problem areas of drug use patterns and quantify the magnitude of the problem at glance. However, they do not answer why the problem exists. Besides, these indicators do not show whether the drug prescribed comply with diagnosis.

## Conclusion

Most of WHO stated core drug use indicators were not met by the three hospitals included in the study. The average number of drugs prescribed per encounter was slightly above the WHO recommendation. Percentages of antibiotic and injection exposure were found to be very high. The average times spent for consultation and dispensing were very short in all the hospitals involved in the study. A few numbers of drugs were adequately labeled. None of the hospitals had developed its own EDL and STG. The recording system and stock management were very poor, especially in HFSUH. Significant amount of key drugs were out of stock. Having this study as a baseline data, in-depth investigation of drug use (qualitative study) should be designed to probe the underlying causes of the problem in these health institutions. Finally, a multitude of remedial intervention strategies (managerial, educational, regulatory and economical strategies) should be designed to reverse the existing problem and modernize the drug utilization patterns on these public health institutions and the Ethiopian health care system in general.

## Abbreviations

DRH: Dilchora Referral Hospital
EDL: Essential drug List
GUH: Gondar University Hospital
HFSUH: Hiwot Fana Specialized University Hospital
HUTRH: Hawassa University Teaching and referral Hospital
JUSH: Jimma University Specialized Hospital
KGH: Karamara General Hospital
RDU: Rational drug use
STG: Standard Treatment Guidelines
WHO: World Health Organization
